# Subcellular localization of HMGB1 in human cholangiocarcinoma: correlation with tumor stage

**DOI:** 10.1007/s12672-021-00446-1

**Published:** 2021-11-08

**Authors:** Nattawan Suwannakul, Kaoru Midorikawa, Chunping Du, Ya-Peng Qi, Jie Zhang, Bang-De Xiang, Mariko Murata, Ning Ma

**Affiliations:** 1 Department of Environmental and Molecular Medicine, Mie University Graduate School of Medicine, 2-174, Edobashi, Tsu, Mie 514-8507 Japan; 2 Department of Pathology, Guangxi Medical University Cancer Hospital, Guangxi, China; 3 Department of Hepatobiliary Surgery, Guangxi Medical University Cancer Hospital, Guangxi, China; 4 Graduate School of Health Science, Suzuka University of Medical Science, 1001-1, Kishioka, Suzuka, Mie 510-0293 Japan

**Keywords:** Cholangiocarcinoma, Subcellular localization, HMGB1, SOX9, YAP1

## Abstract

Cholangiocarcinoma (CCA) is a malignant disease with a poor prognosis, and several studies have been conducted using different molecular markers as a tool for CCA diagnosis, including *Clonorchis sinensis* (CS)-CCA. We initially identified the expression profiles of the three markers of interest, HMGB1, SOX9, and YAP1, using GSE (GSE76297 and GSE32958) datasets. Upregulated levels of these three proteins were detected in CCA samples compared to those in normal samples. To clarify this issue, 24 human CCA tissues with paired adjacent normal tissues were evaluated using immunohistochemical staining. Of the three markers, the total cellular staining intensities were scanned, and subcellular localization was scored in the nuclear and cytoplasmic regions. The intensities of HMGB1, SOX9, and YAP1 were elevated in CCA tissues than the adjacent normal tissues. Individual scoring of subcellular localization revealed that the expression levels of HMGB1 (nucleus) and YAP1 (nucleus and cytoplasm) were significantly different from the pathologic M stage. Moreover, the translocation pattern was categorized using “site-index”, and the results demonstrated that the overexpression of HMGB1 and SOX9 was mostly observed in both the nucleus and cytoplasm, whereas YAP1 was predominantly expressed in the cytoplasm of tumor cells. Interestingly, the site index of HMGB1 was moderately correlated with the tumor stage (r = 0.441, *p* = 0.031). These findings imply that the overexpression of subcellular HMGB1 could be associated with the metastatic status of patients with CS-CCA, which was shown to be effective for CS-CCA prognosis.

## Introduction

Cholangiocarcinoma (CCA) is the second most frequent primary liver tumor globally, with a continually increasing incidence, and is known as a silent killer due to its non-specific symptoms with late diagnosis and few therapeutic options [1]. Despite the various reported risk factors for CCA, liver fluke infestation is a risk factor with strong evidence of rising incidence and mortality rates. In Eastern Asia, the two most common parasitic liver flukes in humans are *Opisthorchis viverrini* and *Clonorchis sinensis*, classified as group 1 human carcinogens by the International Agency for Research on Cancer [2]. The endemic areas of *Clonorchis sinensis* (CS) include Korea, China, Taiwan, and Vietnam, demonstrating high prevalence with active transmission [3]. Due to the clinical silence resulting in poor prognosis, the clarification of potential markers underlying CCA development may help to improve the prognosis of CCA.

High mobility group box 1 (HMGB1) is a nuclear binding protein that acts as an extracellular pro-inflammatory cytokine that has been implicated in high-level expression in several malignancies [4]. Furthermore, in HMG-box family proteins, a canonical HMGB1 protein has a distinctly related DNA-binding domain regulating gene expression with a Sry-related high mobility group box 9 (SOX9) protein using distinct mechanisms [5]. The transcription factor SOX9 plays a vital role in embryonic development and is highly expressed in various tumors, such as prostate, non-small cell lung, and breast cancers [6–8]. Overexpression of SOX9 in cancers is associated with the regulation of cell proliferation and metastasis [9]. Furthermore, SOX9 has been related to tumorigenesis through the Hippo-YAP signaling pathway regulating tissue homeostasis and stem cell self-renewal. SOX9 acts as a downstream target of Yes-associated protein 1 (YAP1), which could enhance epithelial-mesenchymal transition (EMT) in gastric cancer [10] and induce cancer stem cell properties in esophageal cancer [11] through the Hippo-YAP pathway. Likewise, the expression of YAP1 has been associated with poor prognosis in patients with CCA [12]. Several previous studies have reported the characters of these markers by the expression levels and biological functions in various tumors including intracellular CCA (iCCA). By their roles in carcinogenesis, as a proinflammatory cytokine of HMGB1 [13], and the stemness regulators of SOX9 [14] and YAP1 [15], increase a potential for CCA development exhibiting high expression levels in iCCA. However, there has been no study so far to investigate the expressions in parasitic-infected CCA, such as CS-CCA. Therefore, this study aimed to clarify their expressions and correlation in CS-CCA progression.

The gene expression profile has been recently developed and used to identify differentially expressed genes (DEGs) and related pathways during ongoing disease, including cancer. In large global cohorts, Gene Expression Omnibus (GEO) has collected clinical data from patients with malignancies, providing comprehensive cancer genomic sequencing and library of gene expression, which publish accurate predictions for cancer diagnosis, monitoring, molecular typing, and chemotherapy [16]. Here, we identified the expression of candidate markers including HMGB1, SOX9, and YAP1, implicated in the cancer development of patients with CCA using genomic datasets from GEO. We performed immunofluorescence staining for the tissues of patients with CS-CCA, including the tumor and adjacent normal tissues. Additionally, the potential markers were further assessed for their subcellular localization, and correlation with clinical parameters.

## Materials and methods

All methods were performed in accordance with the relevant guidelines and regulations.

### Dataset collection and data analysis

Gene expression profiles of CCAs were obtained from the GEO (https://www.ncbi.nlm.nih.gov/geo/). Using the GEO database, “cholangiocarcinoma” was used as a keyword, and the data of non-tumor and CCA samples were analyzed. In the datasets, 185 samples of GSE76297 (platform GPL17586) and 23 samples of GSE32958 (platform GPL6244) were used to assess DEGs using the GEO2R online analysis tool.

### Human tissue collection

A total of 24 CCA and adjacent tissues were collected from patients who were diagnosed with CS-CCA at Guangxi Medical University Cancer Hospital, China. The clinical parameters including age, sex, TNM classification and stage groups are shown in Table 1. All tissues were acquired from patients who provided written informed consent. This study was approved by the Ethics Committee for Human Research from the Suzuka University of Medical Science, Japan (No. 310).

**Table 1:** The clinical parameters and immunofluorescence (IF) score of the potential markers

Parameters		n	HMGB1		SOX9		YAP1	
			C	N	C	N	C	N
Age	≤ 50	8	2.4 ± 1.5	2.1 ± 1.7	1.9 ± 0.6	1.9 ± 1.5	2.0 ± 1.1	0.3 ± 0.5
	> 50	16	2.9 ± 1.1	2.3 ± 1.9	2.3 ± 0.7	1.9 ± 1.7	2.5 ± 1.0	0.2 ± 0.4
Gender	Male	12	2.5 ± 1.2	2.3 ± 1.8	2.1 ± 0.7	2.4 ± 1.4	2.4 ± 1.1	0.1 ± 0.3
	Female	12	3.0 ± 1.2	2.2 ± 1.9	2.3 ± 0.8	1.4 ± 1.7	2.3 ± 1.0	0.3 ± 0.5
T	T0–T2	14	2.4 ± 1.4	1.9 ± 1.7	2.1 ± 0.8	2.1 ± 1.8	2.5 ± 1.0	0.3 ± 0.5
	T3–T4	10	3.2 ± 0.8	2.6 ± 1.9	2.2 ± 0.6	1.7 ± 1.4	2.1 ± 1.0	0.1 ± 0.3
N	N0	19	2.7 ± 1.3	2.3 ± 1.8	2.2 ± 0.7	1.9 ± 1.7	2.4 ± 1.1	0.2 ± 0.4
	N1	5	3.0 ± 1.0	2.0 ± 1.9	2.0 ± 0.7	2.0 ± 1.6	2.2 ± 0.8	0.2 ± 0.4
M	M0	22	2.7 ± 1.2	2.4 ± 1.7	2.1 ± 0.7	1.9 ± 1.6	2.3 ± 1.0	0.2 ± 0.4
	M1	2	3.5 ± 0.7	0.0 ± 0.0*	3.0 ± 0.0	2.0 ± 2.8	3.0 ± 0.0*	0.0 ± 0.0*
Stage groups	0-I-II	9	2.2 ± 1.5	2.3 ± 1.6	2.1 ± 0.7	2.2 ± 1.7	2.6 ± 1.1	0.3 ± 0.5
	III–IV	15	3.1 ± 0.8	2.1 ± 1.8	2.2 ± 0.7	1.7 ± 1.4	2.2 ± 0.9	0.1 ± 0.3

TNM classification is based on the pathological staging of T: tumor; N: node; M: metastasis

Data are presented as mean ± SD. Statistical different is compared between group of parameter in each target location and * indicates statistically significant (p < 0.05)

*C* cytoplasm, *N* nucleus, *HMGB1* high mobility group box 1, *SOX9* Sry-related high mobility group box 9, *YAP1* yes-associated protein 1

### Immunofluorescence staining (IF)

Protein expression levels of cancer markers including HMGB1, SOX9, and YAP1, were measured in 24 human tissue samples of diagnosed CCA with adjacent tissues. The general histology of the tissue was stained using immunohistochemical technique, followed by hematoxylin and eosin (H&E) staining. Briefly, paraffin-embedded tissues were deparaffinized in xylene and rehydrated in serial alcohol solutions. Antigen retrieval was then performed using a 500 W microwave for 5 min, and non-specific binding protein was blocked by incubation in 1% skim milk. Tissue samples were stained using primary antibodies against HMGB1 (ab18256, Abcam, Cambridge, UK), SOX9 (ab185966, Abcam) and YAP1 (ab52771, Abcam) at a 1:200 dilution, overnight. After washing with phosphate buffer saline (PBS), donkey anti-rabbit IgG Alexa Flour 594 secondary antibody (A11012, Invitrogen, Waltham, MA, USA) at 1:400 dilution in PBS was incubated for 2 h. Stained tissues were mounted using DAPI-fluoromount-G (Southern Biotech, Birmingham, AL, USA) and observed under a fluorescence microscope (Olympus, Tokyo, Japan). The intensity was evaluated in four different areas of each sample using ImageJ software (NIH, USA) for target molecules and DAPI, and the intensity ratio was calculated in comparison to that of the nuclear staining of DAPI, as a reference for the adjustment of cell number. The staining score was observed and recorded as 0–4 by no staining (score 0), weak staining (score 1), moderate staining (score 2), strong staining (score 3), and very strong staining (score 4) in the nuclear and cytoplasmic parts. The total score was the sum of the nuclear and cytoplasmic staining scores. Following the observation, the stained slides were rinsed in distilled water for several hours to remove the cover glass and mounting medium. The clear sample tissues were stained with hematoxylin for 10 min. After washing in tap water for 5 min, tissues were subsequently stained with 1% eosin for 20 s and immediately rinsed in tap water. The mounting medium, malinol (Muto pure chemicals, Tokyo, Japan), was then mounted on tissue sample and allowed to air dry. The histological change was observed under a light microscope (Olympus, Japan).

### Statistical analysis

Statistical tests were conducted using SPSS version 23.0 Software (IBM, NY, USA). For the comparison between tumor and the adjacent tissues, a paired Student’s t-test was used. For comparison between two groups, unpaired Student’s t-test or Welch’s t-test was used, according to equal variance or non-equal variance between groups. The correlation between the index of potential markers and TNM classification was analyzed using the Pearson’s correlation coefficient. Results were considered statistically significant at p < 0.05.

## Results

### The mRNA expression of the potential markers in CCA using the GSE databases

**Fig. 1:**
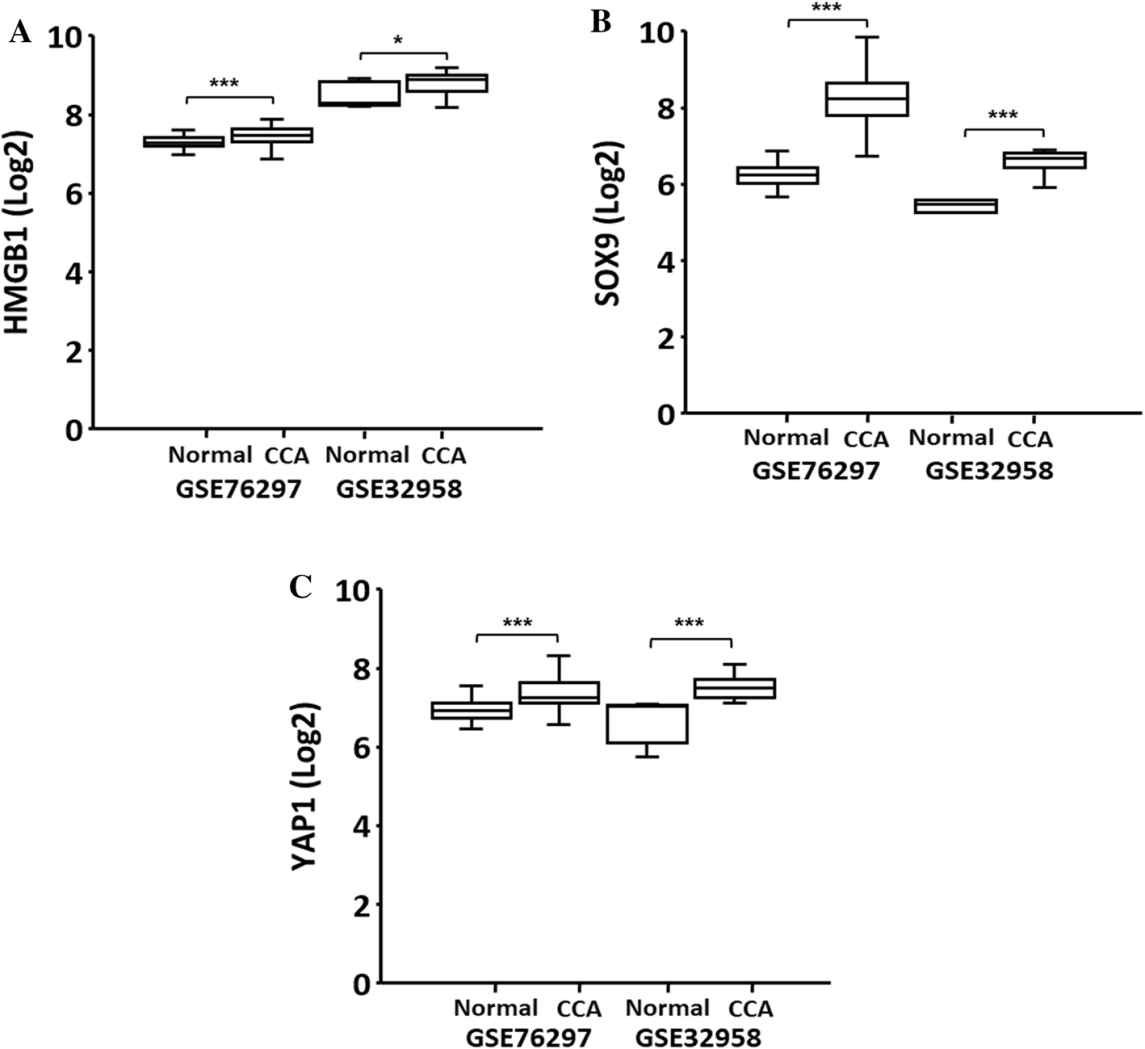
The data of open-access database analysis tools including Gene Expression Omnibus (GEO) dataset (**A**–**C**). The GSE76297; normal tissues (n = 93) and cholangiocarcinoma (CCA) (n = 92), the GSE32958; normal tissues (n = 7) and CCA (n = 16)

To determine the expression levels of HMGB1, SOX9 and YAP1 in CCA samples, we initially analyzed mRNA expression by comparing CCA tissues and normal tissues from the GSE datasets (GSE76297; n = 185 and GSE32958; n = 23), downloaded from the portal database*.* Both of the selected GEO datasets demonstrated the significance upregulation of these three genes in cancer when compared to that of the normal counterpart (Fig. 1A–C). The results indicated that the relative expression of HMGB1, SOX9, and YAP1, was potentially overexpressed in CCA compared to normal tissues.

### The expression and localization of the potential markers in CS-CCA patients

**Fig. 2:**
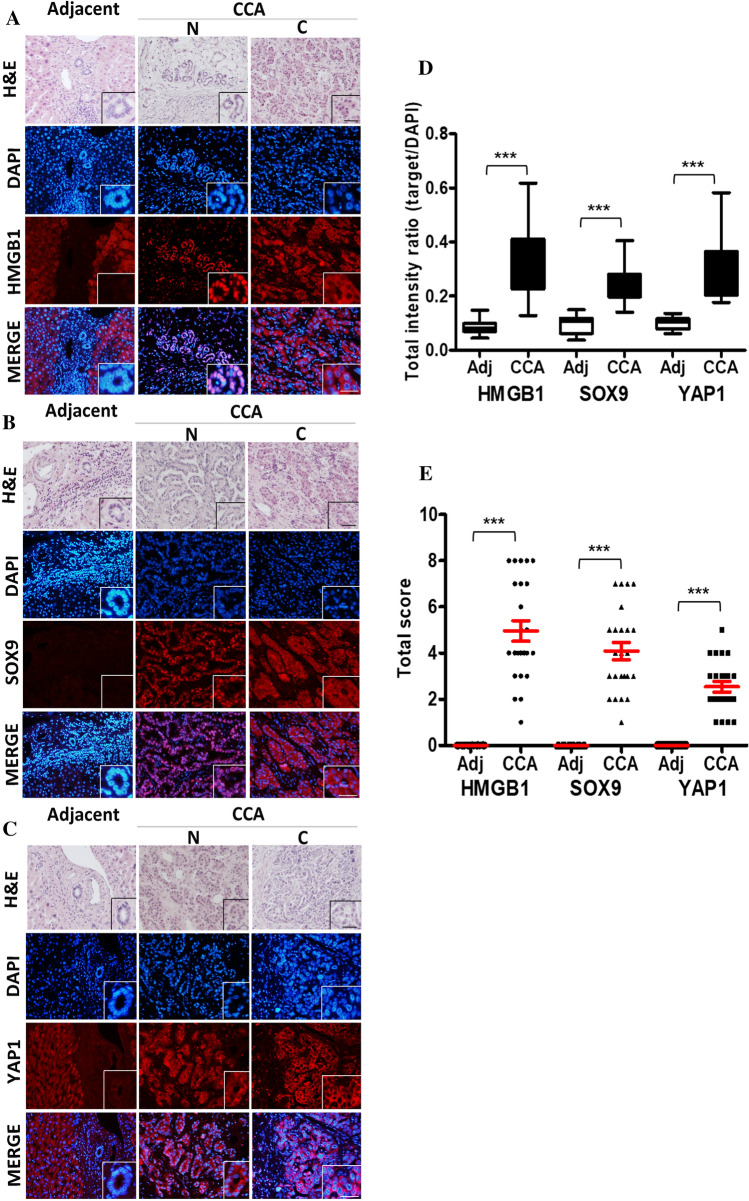
Hematoxylin and eosin (H&E) and immunofluorescence (IF) staining of human *Clonorchis sinensis*-infested cholangiocarcinoma (CS-CCA) and the adjacent normal tissues (bile duct cells) with three potential markers including high mobility group box 1 (HMGB1) (**A**), Sry-related high mobility group box 9 (SOX9) (**B**), and Yes-associated protein 1 (YAP1) (**C**). Typical images of nuclear (N) and cytoplasmic (C) localizations of CCA. The IF intensity ratio between target and DAPI (**D**). IF score of total cellular staining in CS-CCA and the adjacent normal tissues (**E**)

Figure 2 illustrates the histological staining by H&E and immunofluorescence staining of HMGB1 (Fig. 2A), SOX9 (Fig. 2B), and YAP1 (Fig. 2C) in CS-CCA tissues and adjacent normal tissues. There was no or little staining of three markers in bile duct cells of adjacent normal tissues. In CCA tissues, the target molecules were observed in nucleus and/or cytoplasm of cancer cells. Typical nuclear localization pattern (N) and cytoplasmic localization pattern (C) were shown (target molecules in red). When the target molecules localized in nucleus, the merged image with DAPI (nuclear staining in blue) were in magenta (red/blue merge). We examined the protein expression levels of HMGB1, SOX9, and YAP1 by immunofluorescence (IF) staining using IF intensity and score as described in Materials and Methods. The IF intensities of the potential targets in CCA tissues were significantly higher than those in bile duct cells of adjacent normal tissues (Fig. 2D). We evaluated the score individually in the nucleus and cytoplasm, and the sum as the total score. Figure 2E shows the difference in total score by exhibiting significantly higher expression levels of the three proteins in CS-CCA tissues than in adjacent normal tissues, similar to the evaluation of IF intensities (Fig. 2D).

To gain a better understanding of the association between expression level and clinicopathological parameters, we evaluated these alterations using a conventional score (nuclear or cytoplasmic staining), as shown in Table 1. Metastasis (M1) exhibited significantly lower nuclear scores of HMGB1 (*p* = 0.000) and YAP1 (*p* = 0.021), a significantly higher cytoplasmic score of YAP1 (*p* = 0.003), and no significant but higher cytoplasmic score of SOX9 (*p* = 0.079). Thus, the translocation of these molecules from the nucleus to the cytoplasm may be related to prognosis. These findings indicate that these potential markers were strongly overexpressed in CS-CCA tissues by a divergent pattern of localization in tumor cells.

To evaluate the translocation, we established a score of subcellular localization “site-index”, as presented in Table 2. When the staining location was positive only in the nucleus, the site index was “0”, positive in both nucleus and cytoplasm, as “1”, and positive only in the cytoplasm as “2”. In CS-CCA tissues, HMGB1 was expressed only in the nucleus (site index = 0) in 2 cases (8%), both the nucleus and cytoplasm (site index = 1) in 14 cases (58%), and only in the cytoplasm (site index = 2) in 8 cases (33%). SOX9 was mainly expressed in both the cytoplasm and nucleus (16 cases, 67%), and only in the cytoplasm (8 cases, 33%). Strong intensity of YAP1 was found in the cytoplasm (19 cases, 79%) and was occasionally expressed both in the nucleus and cytoplasm (5 cases, 21%).

**Table 2:** Classification of positive staining region in human *Clonorchis sinensis*-infested cholangiocarcinoma (CS-CCA) tissues as “site-index” score

Staining region	Site-index (score)	HMGB1	SOX9 (%)	YAP1 (%)
		Number of patients (%)	Number of patients (%)	Number of patients (%)
Only nucleus	0	2 (8)	0 (0)	0 (0)
Both nucleus and cytoplasm	1	14 (58)	16 (67)	5 (21)
Only cytoplasm	2	8 (33)	8 (33)	19 (79)

*HMGB1* high mobility group box 1, *SOX9* Sry-related high mobility group box 9, *YAP1* yes-associated protein 1

### The correlation of site-index with clinical parameters

To consider the correlation between the expression of the potential markers and known TNM staging, Pearson’s correlation coefficient was additionally performed for analysis. The results revealed that there was a moderate correlation between stage groups and HMGB1 expression using the site-index scoring system (r = 0.441, p = 0.031, Table 3).

**Table 3:** The correlation between the site-index of the potential markers and TNM classification

		TNM classification			
		T	N	M	Stage
HMGB1	Coefficient	0.058	0.129	0.380	0.441*
Site-index	p-value	0.789	0.547	0.067	0.031
	n	24	24	24	24
SOX9	Coefficient	-0.065	-0.145	0.107	0.065
Site-index	p-value	0.764	0.499	0.620	0.764
	n	24	24	24	24
YAP1	Coefficient	0.005	0.011	0.155	0.192
Site-index	p-value	0.983	0.961	0.471	0.368
	n	24	24	24	24

TNM classification is based on the pathological staging of T: tumor; N: node; M: metastasis

Data are presented as a value of coefficient (above) and p-value (below). Statistical different is compared among localization by site-index and * indicates statistically significant (p < 0.05)

*HMGB1* high mobility group box 1, *SOX9* Sry-related high mobility group box 9, *YAP1* yes-associated protein 1

## Discussion

Based on genomic datasets, the expression profiles of candidate genes in CCA cases were analyzed using GEO databases and the increasing levels of feasible tumor markers including HMGB1, SOX9, and YAP1 in patients with CCA. We assumed that the high expression of these three potential markers of CCA could be used for patients with CS-CCA. Screening potential tumor markers might play a crucial role in the personalized diagnosis and treatment of patients with CS-CCA.

SOX family genes encode transcription factors involved in several cellular processes, such as embryonic development, sex determination, and differentiation [17]. Among them, SOX9 has been reported as an oncogene in various human cancers by enhancing cancer cell proliferation [18]. Carrasco-Garcia found that the overexpression of SOX9 was associated with metastatic status and enhanced self-renewal ability in colon cancer cells through their in vivo study, while the colon tumorigenesis was declined in SOX9 knockdown mice [19]. In intrahepatic CCA, the expression level of SOX9 was correlated with tumor stage, that is, a low level of expression was detected in the early stage of the tumor and the high level was related to tumor invasion [20]. Our results in CS-CCA exhibited increased SOX9 expression in both the nucleus and cytoplasm; moreover, the higher level in the cytoplasm showed a positive tendency regarding metastasis of CS-CCA (pathologic M stage) (p = 0.079). In patients with ductal pancreatic cancer bearing TP53 mutations, SOX9 was overexpressed in the cytoplasm and associated with mortality [21], supporting our results. Thus, in this study, the expression level of SOX9 might be an acceptable marker for tumor progression in patients with CS-CCA.

By signaling pathway of tumorigenesis, the Hippo pathway and its coactivator YAP1 provide a critical role in the regulation of organ size and cell proliferation [22]. During cancer development, YAP1 functions as an oncogene by promoting cancer stem cell-like properties [23]. Zhou et al*.* illustrated the oncogenic function of SOX9 in gastric cancer by enhancing EMT through the Hippo-YAP pathway [10]. These data have been supported by evidence of SOX9 as a downstream target of YAP1 and an indirect upstream suppressor of YAP1 in esophageal squamous cell carcinoma [24]. In contrast, YAP also plays a role as a tumor suppressor in breast cancer [25], while Agostino et al. reported that YAP enhanced the proliferative transcriptional activity of mutant p53 proteins in breast cancer patients with poor prognosis [26]. Moreover, in lung cancer, it could play a dual role as an oncogene and tumor suppressor [27]. Cytoplasmic YAP1 has served as an important indicator for colorectal cancer [28] and a dual cytoplasmic-nuclear pattern in prostate cancer [29], although it is frequently expressed in the nucleus in the cancer environment [30–32]. The predominant cytoplasmic YAP1 was observed in patients with CS-CCA associated with tumor metastasis in our current study. Therefore, the site-specific YAP1 expression and its role may change in each cell type.

In mammalian cells, HMGB1 is a highly conserved nuclear protein implicated in several gene regulations, including inflammation and infection-related genes. Nuclear HMGB1 acts as a tumor suppressor as a nucleosome stabilizer and interacts with p53 to regulate the cells [33]. The translocation of nuclear HMGB1 to the cytosol and extracellular space functions as an inflammatory cytokine during cell injury [34]. HMGB1 has been associated with several hallmarks of cancer, including apoptosis, angiogenesis, invasion, and metastasis such as colon, breast, lung, and prostate cancers [35]. Patients with acute-on-chronic liver failure (ACLF) infected by hepatitis B virus showed nuclear-cytoplasmic translocation of HMGB1 in cholangiocytes and were predominantly observed in the cytoplasm, and that re-localization and accumulation of HMGB1 in the cytoplasm is an essential step for its extracellular release which contribute to the inflammatory response during ACLF [36]. This evidence could support and raise the possibility of cytoplasmic HMGB1 localization in CS-CCA progression. Additionally, co-localization of HMGB1 in both the nucleus and the cytoplasm has been detected in colon cancer [37]. The present study indicates that the activation of nuclear and/or cytoplasmic HMGB1 could affect the stage of the tumor in CS-CCA, suggesting a prognostic tumor marker with the role of an inflammatory indicator.

Collectively, HMGB1, SOX9, and YAP1 were identified as potential markers of nucleus-to-cytoplasm transport. Furthermore, the overexpression of HMGB1, SOX9, and YAP1 and their high expression levels in the cytoplasm were related to the pathological metastatic status and stage groups in patients with CS-CCA. Nonetheless, these results should be interpreted and strengthen by the report with increasing the number of cases. As we mentioned above, literature reviews suggested the relationship between p53 and these markers, and possibly control cellular processes through the interaction with wild type or mutant p53 involved in inflammatory regulation in CS-CCA development. For a better understanding of certain functions and mechanisms of regulation, these potential markers might be essential to elucidate a novel CS-CCA therapeutic approach.

## Data Availability

The datasets generated and/or analysed during the current study are available from the corresponding author on reasonable request.
